# Emotional Freedom Techniques for Anxiety Disorders: A Systematic Review

**DOI:** 10.3390/healthcare13172180

**Published:** 2025-09-01

**Authors:** Seong Hun Choi, Soo-Hyun Sung, Gihyun Lee

**Affiliations:** 1 Department of Anatomy and Histology, College of Korean Medicine, Daeggu Haany University, Gyeongsan 38610, Republic of Korea; ck0190@hanmail.net; 2 Department of Policy Development, National Institute for Korean Medicine Development, Seoul 04516, Republic of Korea; 3 College of Korean Medicine, Dongshin University, Naju 58245, Republic of Korea

**Keywords:** EFT, emotional freedom techniques, anxiety, randomized controlled trial, clinical evidence, complementary therapy

## Abstract

Background/Objectives: This systematic review evaluated the efficacy and safety of Emotional Freedom Techniques (EFT) for anxiety disorders, compared with conventional and alternative therapies. Methods: A comprehensive literature search was conducted across nine electronic databases up to February 2025, including only parallel-group randomized controlled trials (RCTs) that investigated EFT as a standalone intervention for anxiety symptoms. The methodological quality of the included studies was assessed using the Cochrane Risk of Bias 2.0 tool. Results: Seven RCTs with 506 participants were included. Populations ranged from clinical to non-clinical groups. EFT interventions varied in duration (1–56 sessions), with control groups including no treatment, supportive interviews, cognitive behavioral therapy (CBT), breathing therapy, and progressive muscle relaxation. All six studies comparing EFT to no intervention reported significant reductions in anxiety symptoms in favor of EFT. Compared to active controls, EFT showed similar or superior effects to breathing therapy and muscle relaxation but no significant difference from CBT. Most studies showed “some concerns” in risk of bias, mainly due to self-reported outcomes and lack of blinding. Conclusions: EFT appears to be a promising and safe complementary intervention for reducing anxiety symptoms, with additional benefits for related psychological outcomes. However, methodological limitations and heterogeneity among studies preclude firm conclusions. High-quality RCTs using standardized protocols and objective outcome measures are warranted to confirm these findings and to explore the effectiveness of EFT as an adjunct to conventional therapies.

## 1. Introduction

Anxiety disorders represent some of the most common mental health conditions globally, leading to considerable personal distress, diminished quality of life, and greater demands on healthcare systems [1,2]. The prevalence of anxiety disorders is estimated to be around 7.3%, but this rate surged to approximately 35.1% during the COVID-19 pandemic [3,4]. These disorders are primarily characterized by excessive and persistent fear, anxiety, or avoidance of perceived threats, often manifesting as panic attacks [5]. The spectrum of anxiety disorders includes generalized anxiety disorder, panic disorder, social anxiety disorder, and specific phobias, each presenting distinct clinical features and symptom profiles [6,7]. Frequently, individuals with anxiety disorders experience somatic symptoms such as elevated heart rate, shortness of breath, and muscle tension, which can significantly impair daily functioning. In a study by Russ et al. [8], even mild levels of psychological distress—including anxiety—were significantly associated with increased risk of all-cause mortality in the general population. These findings underscore the necessity of early identification and effective management of anxiety symptoms, regardless of severity, to potentially improve not only mental health but also overall survival outcomes.

Conventional treatment for anxiety disorders typically involves pharmacotherapy and cognitive behavioral therapy (CBT). Pharmacological interventions primarily include selective serotonin reuptake inhibitors (SSRIs), serotonin–norepinephrine reuptake inhibitors (SNRIs), and benzodiazepines [9]. While these medications are generally effective in reducing anxiety symptoms, they are often associated with adverse effects such as headaches, gastrointestinal disturbances, and sleep-related issues. In contrast, CBT is widely recognized as the most effective form of psychotherapy for anxiety disorders and is notably free of the side effects commonly observed with pharmacological treatments [10]. However, CBT can be challenging for some individuals due to limited accessibility, time commitments, and the intensive engagement required from both therapist and patient.

As a result of these limitations, growing interest has emerged in complementary and alternative medicine (CAM) approaches, including natural therapies, meditation, yoga, aromatherapy, music therapy, and Emotional Freedom Techniques (EFT). Among these, EFT has gained particular attention as a hybrid intervention that combines acupressure—based on stimulation of meridian points—with elements of cognitive restructuring [11]. During EFT sessions, individuals focus on specific emotions or traumatic memories while tapping on major meridian points of the body using their fingertips [11]. This approach integrates concepts from traditional medicine with modern psychological techniques, offering a novel and often rapid method for achieving emotional regulation and symptom relief [12].

The proposed mechanisms underlying EFT’s therapeutic effects encompass both psychological processes—such as exposure and cognitive reframing—and physiological regulation through acupressure. Notably, research has identified alterations in biological markers such as cortisol levels and heart rate variability following EFT intervention [13]. EFT has demonstrated efficacy across a wide range of populations, including individuals with chronic health conditions (e.g., fibromyalgia), students, healthcare professionals, and the general public. Moreover, it is adaptable to various delivery formats, including individual, group-based, and virtual platforms [12,13].

Empirical evidence suggests that EFT contributes to improvements in several psychological outcomes, including reduced anxiety and depressive symptoms, decreased post-traumatic stress disorder (PTSD) symptoms, and enhanced self-efficacy [14,15]. Its rapid effect and non-reliance on medication have led to its growing adoption as a CAM for anxiety disorders. Accordingly, the use of EFT has expanded into both clinical and non-clinical domains. Millions of users engage with virtual EFT content through websites, online sessions, and mobile applications annually [16], and its application has been extended to fields such as education, organizational psychology, and sports performance.

In a study conducted by Clond et al. [1] in 2016, EFT was found to be effective in reducing anxiety symptoms compared to both no-treatment and active control conditions. However, since the review included RCTs focusing on PTSD and was conducted nearly a decade ago, an updated systematic review specifically evaluating the effectiveness of EFT for anxiety disorders is warranted. In line with the *Diagnostic and Statistical Manual of Mental Disorders, Fifth Edition* (DSM-5), PTSD is categorized separately under trauma- and stressor-related disorders and is no longer classified as an anxiety disorder [17]. In this review, we examined clinical trials investigating the efficacy of EFT as an alternative intervention for anxiety disorders. This systematic review includes all relevant research published up to February 2025, thereby encompassing studies conducted during the COVID-19 pandemic period. We compared outcomes across studies, evaluated methodological limitations and potential biases, and proposed strategies for enhancing the rigor of future research. Through this, we aim to offer direction for the advancement of EFT-related research in the field of anxiety treatment.

## 2. Materials and Methods

### 2.1. Protocol and Registration

This systematic review was prospectively registered with the International Prospective Register of Systematic Reviews (PROSPERO; Registration No. CRD420251079708). The review process adhered to the guidelines outlined in the Preferred Reporting Items for Systematic Reviews and Meta-Analyses (PRISMA) statement [18] (see Supplementary Materials Table S1 for the checklist).

### 2.2. Data Sources and Searches

A comprehensive literature search was performed across nine electronic databases: MEDLINE via PubMed, EMBASE, Cochrane Central Register of Controlled Trials (CENTRAL), CINAHL Plus, OASIS, KoreaMed, ScienceON, RISS, and the Korea Citation Index (KCI). The search included studies published up to February 2025, with no language restrictions applied.

Search terms included combinations of Medical Subject Headings (MeSH) and keywords: (“emotional freedom technique” OR “tapping therapy”) AND (“anxiety” OR “anxiety disorders”) AND (“clinical trial” OR “randomized controlled trial”). Detailed search strategies for each database are presented in Supplementary Table S2.

### 2.3. Eligibility Criteria

#### 2.3.1. Study Design

Only parallel-group randomized controlled trials (RCTs) were eligible for inclusion. Studies employing crossover designs or lacking randomization were excluded.

#### 2.3.2. Population

We included RCTs involving participants with symptoms of anxiety, regardless of their underlying medical or psychological conditions. No restrictions were imposed based on age, sex, or country. In line with the DSM-5, PTSD is categorized separately under trauma- and stressor-related disorders and is no longer classified as an anxiety disorder [17]; therefore, studies exclusively targeting individuals with PTSD were excluded.

#### 2.3.3. Interventions

Included studies evaluated EFT as the sole therapeutic intervention for anxiety. Trials in which EFT was delivered in combination with other therapeutic modalities were excluded. Only studies implementing EFT in accordance with the second edition of the official EFT Manual were included; studies deviating from the protocol—such as tapping on non-meridian points—were not considered [19].

#### 2.3.4. Comparators

There were no limitations on the nature of control interventions. Acceptable comparators included wait-list controls, breathing exercises, and CBT, among others.

#### 2.3.5. Outcome Measures

The primary outcome was anxiety symptom severity measured by validated psychometric scales, such as the State-Trait Anxiety Inventory (STAI), Revised Children’s Manifest Anxiety Scale (RCMAS), or the Symptom Assessment-45 (SA-45). The STAI is a widely used self-report questionnaire that measures both situational (state) and general (trait) anxiety levels in adults, consisting of separate scales for each dimension. The RCMAS is a validated self-report inventory designed to assess anxiety symptoms in children and adolescents, focusing on physiological anxiety, worry/oversensitivity, and social concerns. The SA-45 is a brief, multidimensional self-report measure evaluating symptoms across nine domains, including anxiety, depression, hostility, and somatization, commonly used for clinical and research purposes.

Secondary outcomes included (1) psychological variables such as depression and anger, (2) self-efficacy, (3) cortisol levels, (4) quality of life, and (5) adverse events.

### 2.4. Study Selection

Two reviewers (S.H.C. and S.-H.S.) independently screened the titles and abstracts of all retrieved records to identify potentially eligible studies. Full texts of selected articles were then reviewed against predefined inclusion criteria. Discrepancies at any stage were resolved through discussion and consensus.

### 2.5. Data Extraction Process

Data from the eligible RCTs were independently extracted by two reviewers (S.H.C. and S.-H.S.) using a standardized data extraction form developed in Microsoft Excel (Microsoft Corp., Redmond, WA, USA). Extracted information included study characteristics (authors, publication year, and country), participant demographics, sample size, details of the EFT and comparator interventions, outcome measures, main findings, and any reported adverse events. Any disagreements were resolved by a third author (G.L.).

### 2.6. Risk of Bias Assessment

The methodological quality of the included RCTs was evaluated using the Cochrane Risk of Bias (ROB) 2.0 tool [20]. This tool assesses potential bias across five domains: randomization process, deviations from intended interventions, missing outcome data, measurement of the outcome, and selection of the reported results. Each domain was rated as “low risk”, “some concerns”, or “high risk”. Two reviewers (S.H.C. and S.-H.S.) independently conducted the assessments, and any disagreements were discussed and resolved with a corresponding author (G.L.).

### 2.7. Data Analysis and Synthesis

Due to significant heterogeneity in control interventions and outcome measures across the included studies, a meta-analysis was not feasible. Therefore, we conducted a narrative synthesis to qualitatively summarize and compare study findings.

## 3. Results

### 3.1. Study Selection and Characteristics

**Figure 1:**
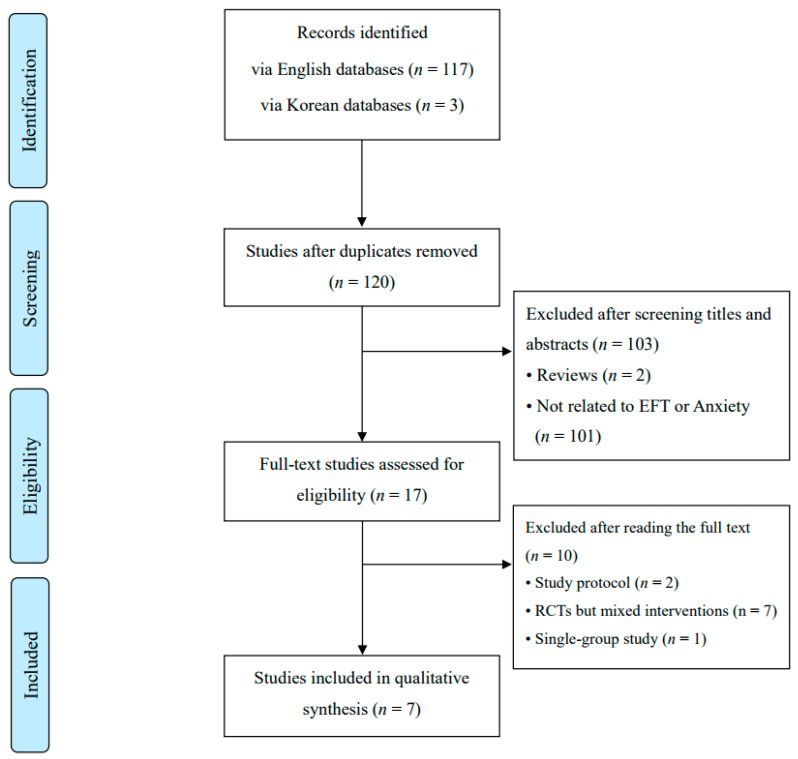
PRISMA flow diagram of the study process. EFT: Emotional Freedom Techniques, PRISMA: Preferred Reporting Items for Systematic Reviews and Meta-Analyses, RCTs: Randomized Controlled Trials.

A total of 120 records were initially identified through database searches (Figure 1). After excluding duplicate entries, review articles, and studies unrelated to either EFT or anxiety, the full texts of 17 articles were assessed for eligibility. Following this screening process, seven RCTs [21,22,23,24,25,26,27] met the inclusion criteria and were included in the final analysis. Sample sizes in these studies ranged from 31 to 120 participants. The EFT interventions varied considerably in both frequency and duration, spanning from a single-session format to daily sessions administered over an eight-week period. Control conditions included no intervention, supportive interviews, CBT, breathing exercises, and progressive muscle relaxation techniques. The seven included studies were conducted in the following countries: Sweden [21], the United States [22,23], Turkey [24,26,27], and South Korea [25]. The characteristics of the seven included studies are summarized in Table 1.

### 3.2. Participants

A total of 506 participants with anxiety symptoms were included across the seven RCTs. Among them, 184 participants were assigned to the EFT intervention group, and 322 to the control groups. Two studies [21,25] involved clinical populations (fibromyalgia and Hwabyung); four studies [22,23,26,27] included non-clinical populations such as students, nurses, and the general public; and one study [24] focused on pregnant women.

### 3.3. EFT Interventions

The number of EFT sessions across the seven studies [21,22,23,24,25,26,27] ranged from 1 to 56, with a mean of 10.7 sessions. The intervention period varied considerably, from a single day to up to 10 months, indicating a wide range in treatment intensity and duration.

### 3.4. Control Interventions

Control conditions in the included trials were categorized into two types. First, six studies [21,22,23,24,26,27] included no-intervention groups. Second, five studies employed active control groups, including supportive interviews [22], CBT [23], breathing therapy [24,27], and progressive muscle relaxation [25]. Notably, four studies [22,23,24,27] included both no-intervention and active control groups for comparison.

### 3.5. Effects of EFT

#### 3.5.1. Primary Outcome

##### Anxiety Outcome

All six studies comparing EFT to no-intervention controls [21,22,23,24,26,27] reported statistically significant reductions in anxiety symptoms in favor of EFT. In one study by Church [22], a single EFT session led to significant improvements in anxiety (*p* < 0.01) compared to supportive interviews. However, in another study [23], three EFT sessions administered over five months did not yield statistically significant differences when compared with CBT.

Two studies [24,27] compared EFT with breathing therapy. In pregnant women [24], EFT significantly reduced anxiety as measured by Wijma Delivery Expectancy/Experience Questionnaire (W-DEQ-B) and Subjective Units of Distress Scale (SUDS) during the active and transition phases of labor (*p* < 0.01). Another study [27] demonstrated that both EFT and breathing therapy significantly reduced anxiety (as measured by STAI, Speech Anxiety Scale (SAS), and SUDS; all *p* < 0.001) compared to no intervention, with EFT showing a larger effect size for speech anxiety (Cohen’s d > 0.8). In the study involving Hwabyung patients [25], four EFT sessions showed comparable effects to progressive muscle relaxation, with no statistically significant difference between the two groups in anxiety outcomes.

#### 3.5.2. Secondary Outcomes

##### Psychological Indicators (Depression and Anger)

Three studies [21,22,25] assessed psychological variables. EFT significantly reduced depression symptoms compared to no intervention in two studies [21,22]. When compared to active controls, one study [22] found a significant difference favoring EFT over supportive interviews, whereas another study [25] reported no significant differences between EFT and progressive muscle relaxation. In Kwak’s study [25], a significant reduction was observed only in trait anger (*p* < 0.05) following EFT, with no difference for other anger dimensions.

##### Self-Efficacy

In the fibromyalgia patient group [21], daily EFT sessions over eight weeks did not lead to a statistically significant improvement in self-efficacy compared to the no-intervention group (*p* = 0.10).

##### Cortisol Levels

Church et al. [22] reported a significant reduction in salivary cortisol following a single EFT session compared to both supportive interviews and no intervention (*p* < 0.05).

##### Quality of Life

Brattberg [21] found statistically significant improvements in five out of eight quality of life domains—including role—physical, vitality, social functioning, role—emotional, and mental health (all *p* < 0.05)—following EFT.

##### Adverse Events

Only three studies [21,23,25] reported adverse events (AEs), and none reported any occurrences in either the intervention or control groups. The remaining four studies [22,24,26,27] did not report on AEs.

### 3.6. Risk of Bias

**Figure 2:**
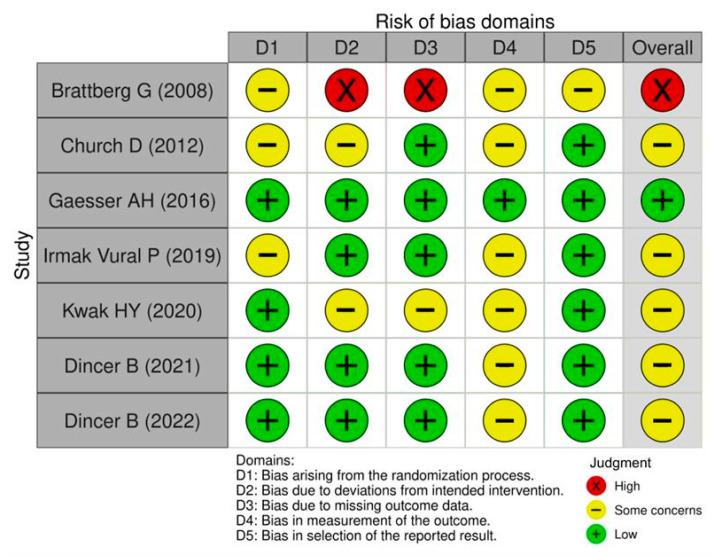
Risk of bias assessment for included clinical studies [21,22,23,24,25,26,27].

Among the seven RCTs included in this review, the overall ROB was judged as “some concerns” in the majority of studies (*n* = 5, 71.4%) (Figure 2). One study was rated as having a low ROB, while another was assessed as high ROB. In five studies [22,24,25,26,27], outcome measurement was rated as “some concerns” primarily due to the lack of assessor blinding and reliance on self-reported data, which may introduce subjective bias. In contrast, one study [23] was assessed as having a low ROB across all domains, owing to its rigorous design, well-implemented intervention, low attrition rate, independent and blinded pre-post assessments, and comprehensive reporting of outcomes.

## 4. Discussion

### 4.1. Main Findings and Implications

This systematic review evaluated the effectiveness and safety of EFT as a treatment for anxiety disorders. Seven RCTs were included. All six studies comparing EFT with no intervention [21,22,23,24,26,27] demonstrated statistically significant improvements in anxiety symptoms in the EFT groups. However, in the only study comparing EFT with CBT [23], no significant difference was observed between the two interventions. In contrast, one trial [24] reported that EFT was significantly more effective than breathing therapy in reducing anxiety symptoms among pregnant women, and another study [27] found that although both EFT and breathing therapy outperformed no intervention, EFT yielded a greater effect size, particularly in reducing speaking anxiety.

The ROB assessment indicated that several included studies exhibited either a high ROB or raised some concerns across key methodological domains, including randomization procedures [21,22,24], deviations from intended interventions [21,22,25], missing outcome data [21,25], outcome measurement [21,22,24,25,26,27], and selective reporting of results [21]. These methodological weaknesses may have introduced performance and detection biases, potentially inflating the estimated effects of EFT on anxiety outcomes [28]. As such, the findings of this review should be interpreted with caution, considering the possible influence of these limitations on the reported effectiveness of EFT. To build a more robust evidence base, future studies should employ rigorous randomized controlled trial designs with methodological safeguards to minimize bias.

Over the past two decades, the field of EFT research has undergone significant methodological refinement. Early studies were often constrained by small sample sizes, heterogeneous intervention protocols, and the absence of rigorous control conditions, thereby limiting both cross-study comparability and the generalizability of results [12]. In contrast, more recent investigations—including several highlighted in this review—have increasingly implemented standardized EFT protocols within well-powered, parallel-group randomized controlled trials [29]. This shift toward greater methodological rigor facilitates meta-analytic integration and enhances the alignment of EFT research with evidence-based medical standards. Furthermore, contemporary trials have begun to focus on clinically relevant populations, incorporate active control groups (e.g., breathing therapy or progressive muscle relaxation), and utilize validated, standardized outcome measures. Collectively, these advancements reflect a broader movement toward enhanced scientific validity and reproducibility within EFT research. As such, EFT is approaching a level of methodological consistency that supports its potential integration into conventional healthcare systems, including clinical guideline development, insurance reimbursement frameworks, and interdisciplinary treatment models.

In South Korea, EFT was officially recognized as a new medical technology in 2017 and has been listed as a non-reimbursed intervention under the National Health Insurance system for the treatment of PTSD [30]. This recognition suggests that the Korean government acknowledges EFT as a standardized clinical procedure with therapeutic potential for PTSD. Although currently not reimbursed, such designation represents a transitional phase before full insurance coverage. With further evidence, EFT may eventually be covered by national insurance, allowing broader access. Given that anxiety disorders are more prevalent than PTSD and have a high potential for clinical application, the need for additional research is evident.

### 4.2. Limitations and Strengths

A notable strength of this systematic review is the inclusion of studies that employed a relatively standardized EFT protocol, enhancing its credibility as an evidence-based therapeutic option. This review is subject to several limitations. One notable issue is the considerable heterogeneity in the number and duration of EFT sessions across the included studies. For instance, one study [21] delivered an extensive protocol consisting of 56 online self-help sessions over an 8-week period—an approach that markedly deviates from the intervention formats used in other trials. In contrast, the majority of studies implemented substantially shorter protocols, with the total number of EFT sessions ranging from one to nine, typically administered over briefer time frames (e.g., a single session or weekly sessions over four weeks). This variability limits direct comparison across studies and suggests that findings from shorter interventions may not be generalizable to those employing prolonged or intensive EFT protocols. Secondly, due to heterogeneity in anxiety measurement tools and reporting formats, a meta-analysis could not be conducted. Future research should adopt standardized outcome measures for anxiety and provide detailed statistical data (e.g., mean values, standard deviations, confidence intervals) to facilitate quantitative synthesis. Ideally, these data should be made available in supplementary materials to enhance the reproducibility and utility of future meta-analyses. Thirdly, only one study was rated as having a low overall ROB, while the remaining six studies were assessed as having “some concerns”, particularly in the outcome measurement domain. This issue stems from the exclusive use of self-reported scales for anxiety assessment, which are subject to potential measurement bias [31]. Incorporating objective biological markers such as salivary cortisol, serum serotonin, or inflammatory markers (e.g., IL-6, TNF-α) may enhance the reliability and validity of outcome assessments [32,33,34].

### 4.3. Future Perspectives

In clinical practice, anxiety disorders are typically addressed through a multimodal approach that combines pharmacotherapy, psychotherapy (e.g., CBT), lifestyle modifications, and behavioral interventions tailored to the patient’s condition [35]. Thus, future studies should investigate the effectiveness of EFT as an adjunct to conventional therapies and identify optimal combination strategies. This review included RCTs published up to February 2025, encompassing studies conducted during the COVID-19 pandemic. Although none of the included trials specifically targeted individuals diagnosed with COVID-19, a single-arm clinical study by Tambunan et al. [36] investigated the effects of EFT in this population. The study reported statistically significant reductions in both anxiety and depression following the intervention. These preliminary findings suggest that EFT may hold potential in managing psychological distress among COVID-19 patients. Further research is warranted to strengthen the clinical evidence base and explore the applicability of EFT across diverse contexts and conditions.

The inherent flexibility of EFT supports its integration across a wide range of delivery modalities, including mobile applications, digital health platforms [37,38], group-based programs, self-help formats [22], and as an adjunctive intervention within psychotherapeutic settings [39]. EFT has also been implemented in various institutional contexts such as medical, educational, workplace, and correctional environments [8]. In particular, the increased adoption of telehealth and digital platforms during and after the COVID-19 pandemic has expanded the accessibility and scalability of EFT beyond traditional clinical settings [38]. This diversity in application underscores the method’s broad generalizability and adaptability, positioning it as a potentially valuable tool for both treatment and prevention of anxiety-related symptoms across heterogeneous populations. Moving forward, research should aim to optimize EFT delivery for each modality, identify the specific subgroups that benefit most, and develop standardized protocols to facilitate its integration into comprehensive, multidisciplinary approaches to anxiety management.

## 5. Conclusions

This systematic review evaluated the efficacy and safety of EFT for anxiety disorders by conducting a comprehensive search of nine electronic databases. Seven RCTs comprising 506 participants met the inclusion criteria. The study populations comprised both clinical groups—including individuals with fibromyalgia, pregnant women, and patients with Hwabyung—and non-clinical cohorts, such as students and nurses. The intensity of interventions ranged from a single session to as many as 56 sessions, with control groups including no treatment, supportive counseling, CBT, breathing exercises, and progressive muscle relaxation.

Compared to no-treatment controls, six studies demonstrated that EFT significantly reduced anxiety symptoms. When evaluated against active controls, EFT showed comparable or superior benefits relative to breathing exercises and progressive muscle relaxation, while no significant difference was observed compared to CBT. Importantly, no serious adverse events were reported across the studies. Nevertheless, most trials raised some concerns regarding ROB, largely due to reliance on self-reported measures and limited assessor blinding. The heterogeneity in populations, interventions, and outcome assessments precluded a formal meta-analysis. Overall, EFT appears to be a safe and rapidly effective complementary intervention for anxiety reduction. However, further rigorously designed, adequately powered RCTs with standardized protocols and objective outcome measures are needed to confirm its comparative effectiveness and clarify its role alongside conventional treatments.

## Figures and Tables

**Table 1:** Characteristics of the included studies.

First Author (Year) Country	Patients’ Disease, Gender (M/F), Age (M ± SD), Sample Size (Randomized/Analyzed)	EFT Intervention	Control Intervention	Outcome Measurements	Main Results (Between-Group Difference, Mean ± SD)	Adverse Events
Brattberg G (2008) Sweden [21]	Fibromyalgia women with anxiety Gender: 0/62 Age: 43.8 ± 8.8 Sample: 86/62	EFT, *n* = 26, total 56 sessions (once a day for 8 weeks)	No intervention, *n* = 36	Anxiety scale (HADS) (1) Anxiety (2) Depression Quality of life (SF-36) (1) Physical functioning (2) Role—physical (3) Bodily pain (4) General health (5) Vitality (6) Social functioning (7) Role—emotional (8) Mental health Self-efficacy scale (GSE)	1. (1) *p* < 0.05, (E) 7.4 ± 4.5, (C) 9.7 ± 5.5 (2) *p* < 0.05, (E) 6.9 ± 4.4, (C) 9.1 ± 5.1 2. (1) NS (*p* = 0.30), (E) 53.4 ± 16.3, (C) 46.1 ± 18.8 (2) *p* < 0.01, (E) 35.6 ± 32.5, (C) 18.8 ± 29.5 (3) NS (*p* = 0.20), (E) 38.6 ± 17.4, (C) 26.6 ± 15.6 (4) NS (*p* = 0.09), (E) 46.7 ± 19.1, (C) 37.5 ± 23.2 (5) *p* < 0.05, (E) 35.8 ± 21.7, (C) 18.1 ± 16.4 (6) *p* < 0.05, (E) 55.8 ± 22.9, (C) 42.7 ± 25.1 (7) *p* < 0.05, (E) 65.4 ± 40.5, (C) 47.2 ± 46.0 (8) *p* < 0.05, (E) 66.9 ± 20.8, (C) 58.2 ± 21.9 3. NS (*p* = 0.10), (E) 31.6 ± 4.8, (C) 28.6 ± 6.2	(E) No AEs occurred (C) No AEs occurred
Church D (2012) United States [22]	Non-clinical people with anxiety Gender: 17/66 Age: 51.3 ± 12.02 Sample: 83/83	EFT (*n* = 28), total 1 session	(C1) Supportive interviews, *n*= 28, total 1 session (C2) No intervention, *n* = 27	Symptom assessment-45 (1) Anxiety (2) Depression (3) Hostility (4) Interpersonal sensitivity (5) Obsessive–compulsive (6) Paranoid ideation (7) Phobic anxiety (8) Psychoticism (9) Somatization (10) PST (11) GSI Salivary cortisol level	1. (1) *p* < 0.001, (E) 49.25 ± 0.9, (C1) 55.94 ± 0.9, (C2) 54.44 ± 0.94 (2) *p* < 0.01, (E) 51.05 ± 0.72, (C1) 54.59 ± 0.73, (C2) 54.67 ± 0.75 (3) *p* < 0.05, (E) 54.26 ± 0.53, (C1) 56.2 ± 0.54, (C2) 55.97 ± 0.55 (4) *p* < 0.001, (E) 50.93 ± 0.59, (C1) 53.88 ± 0.61, (C2) 54.17 ± 0.62 (5) *p* < 0.01, (E) 51.32 ± 0.96, (C1) 55.77 ± 0.98, (C2) 55.58 ± 0.99 (6) *p* < 0.001, (E) 48.51 ± 0.76, (C1) 53.01 ± 0.77, (C2) 52.76 ± 0.77 (7) NS (*p* = 0.51), (E) 59.84 ± 0.48, (C1) 60.58 ± 0.48, (C2) 60.45 ± 0.49 (8) *p* < 0.05, (E) 58.97 ± 0.35, (C1) 59.78 ± 0.35, (C2) 60.51 ± 0.36 (9) *p* < 0.01, (E) 53.22 ± 0.9, (C1) 54.26 ± 0.9, (C2) 57.36 ± 0.93 (10) *p* < 0.001, (E) 47.19 ± 0.85, (C1) 53.21 ± 0.87, (C2) 53.71 ± 0.89 (11) *p* < 0.001, (E) 46.62 ± 0.88, (C1) 52.56 ± 0.9, (C2) 53.48 ± 0.93 2. *p* < 0.05, not reported	n.r.
Gaesser AH (2016) United States [23]	Students with anxiety Gender: 18/45 Age: 10–18 Sample: 63/62	(E) EFT, *n* = 20, total 3 sessions (5 months)	(C1) Cognitive Behavioral Therapy, *n* = 21, total 3 sessions (5 months) (C2) No intervention, *n* = 21	Anxiety scale (RCMAS)	(E) 52.16 ± 9.23, (C1) 54.82 ± 5.81, (C2) 57.93 ± 6.02, (E vs C1) NS (*p* = 0.18), (E vs C2) *p* < 0.01, (C1 vs C2) NS (*p* = 0.12)	(E) No AEs occurred (C) No AEs occurred
Irmak Vural P (2019) Turkey [24]	Pregnant women with anxiety Gender: 0/120 Age: (E) 27.29 ± 3.97 (C1) 27.51 ± 4.65 (C2) 27.36 ± 4.19 Sample: 120/120	(E) EFT (*n* = 35), total 9 sessions (latent phase; the intervention was delivered three times during each of the active and transition phases)	(C1) Breathing Therapy (*n* = 35), total sessions: n.r. (The intervention was administered as needed whenever uterine contractions occurred) (C2) No intervention (*n* = 50)	Anxiety scale (1) W-DEQ-A (2) W-DEQ-B (3) SUDS in latent phase (4) SUDS in active phase (5) SUDS in transition phase	1. (1) NS (*p* = 0.861), (E) 56.40 ± 16.20, (C1) 54.34 ± 12.84, (c2) 55.16 ± 17.43 (2) *p* < 0.01, (E) 59.17 ± 18.52, (C1) 59.57 ± 18.76, (c2) 71.74 ± 13.74 (3) NS (*p* = 0.055) between (E) 1.91 ± 1.52 and (C1) 2.80 ± 1.81 (4) *p* < 0.001 between (E) 2.51 ± 1.40 and (C1) 4.00 ± 1.48 (5) *p* < 0.001 between (E) 3.86 ± 1.44 and (C1) 5.94 ± 1.78	n.r.
Kwak HY (2020) South Korea [25]	Hwabyung * patients with anxiety Gender: 3/28 Age: (E)50 ± 13.31, (C)49.67 ± 12.20 sample: 40/31	EFT, *n* = 15, total 4 sessions (once a week for 4 weeks)	Progressive muscle relaxation, *n* =16, total 4 sessions (once a week for 4 weeks)	Anxiety scale (STAI) (1) State anxiety (2) Trait anxiety STAXI (1) State anger (2) Trait anger (3) Anger—controlled (4) Anger—out (5) Anger—in Hwabyung scale (1) Character (2) Symptom (3) Core VAS (4) Som VAS (5) Psych VAS BDI	1. (1) NS (*p* = 0.947), (E) 47.73 ± 2.39, (C) 49.44 ± 2.2 (2) NS(*p* = 0.285), (E) 53.33 ± 2.072, (C) 48.75 ± 8.87 2. (1) NS (*p* = 0.627), (E) 18.67 ± 2.12, (C) 21.56 ± 1.81 (2) *p* < 0.05, (E) 24.33 ± 2.1, (C) 25.13 ± 1.66 (3) NS (*p* = 0.087), (E) 20 ± 1.2, (C) 18.56 ± 0.83 (4) NS (*p* = 0.395), (E) 18.53 ± 5.07, (C) 18 ± 1.38 (5) NS (*p* = 0.873), (E) 20.87 ± 0.87, (C) 20.06 ± 0.92 3. (1) NS (*p* = 0.83), (E) 39.2 ± 1.89, (C) 37.75 ± 1.77 (2) NS (*p* = 0.65), (E) 40.6 ± 1.93, (C) 40.94 ± 2.18 (3) NS (*p* = 0.626), (E) 39.59 ± 1.53, (C) 40.44 ± 2.42 (4) NS (*p* = 0.788), (E) 29.07 ± 0.82, (C) 29.08 ± 2.08 (5) NS (*p* = 0.691), (E) 29.01 ± 1.27, (C) 27.04 ± 1.35 4. NS (*p* = 0.19), (E) 27 ± 1.65, (C) 26.13 ± 3.01	(E) No AEs occurred (C) No AEs occurred
Dincer B (2021) Turkey [26]	Nurses with anxiety Gender: 8/64 Age: 33.45 ± 9.63 Sample: 80/72	EFT (*n* = 35), total 1 session	No intervention (*n* = 37)	Anxiety scale (1) STAI (2) SUDS Burnout scale	1. (1) *p* < 0.001, (E) 32.25 ± 4.67, (C) 64.43 ± 7.68 (2) *p* < 0.001, (E) 2.85 ± 1.21, (C) 7.40 ± 1.53 2. *p* < 0.001, (E) 2.48 ± 1.06, (C) 3.43 ± 0.76	n.r.
Dincer B (2022) Turkey [27]	Students with anxiety Gender: 8/68 Age: 20.36 ± 0.81 Sample: 78/76	EFT (*n* = 25), total 1 session	(C1) Breathing Therapy (*n* = 26), total 1 session (C2) No intervention (*n* = 25)	Anxiety scale (1) SUDS (2) STAI-1 (3) STAI-2 (4) SAS	1. (1) *p* < 0.001 between (E) 3 (2–4) and (C2) 8 (6–8) *p* < 0.001 between (C1) 6 (5–6) and (C2) 8 (6–8) (2) *p* < 0.001 between (E) 32 (28.5–38) and (C2) 61.04 (57–68.5) *p* < 0.001 between (C1) 52 (45–56) and (C2) 61.04 (57–68.5) (3) NS, (*p* > 0.05), (E) 36 (35–42), (C1) 42 (35–42), (C2) 39.16 (35–42) (4) *p* < 0.001 between (E) 45 (38–52.5) and (C2) 84 (68–86.5) *p* < 0.001 between (C1) 60 (55.75–70.25) and (C2) 84 (68–86.5) **	n.r.

* Hwabyung is a culture-bound syndrome commonly observed in South Korea, characterized by chronic suppressed anger, somatic complaints, and emotional distress. ** The effect size of EFT on speaking anxiety was larger than that of the Breathing Therapy (Cohen d > 0.8). AEs: Adverse Events, BDI: Beck Depression Inventory, (C): Control group, (E): Experimental group, EFT: Emotional Freedom Techniques, GSE: General Self-Efficacy scale, GSI: General Symptom Index, HADS: Hospital Anxiety and Depression Scale, n.r.: not reported, NS: No Significant differences between groups, PST: Positive Symptom Total, RCMAS: Revised Children’s Manifest Anxiety Scale—2, SAS: Speech Anxiety Scale, SF-36: 36-item Short Form health survey, STAI: State-Trait Anxiety Inventory, STAXI: State-Trait Anger Expression Inventory, SUDS: Subjective Units of Distress Scale, VAS: Visual Analogue Scale, W-DEQ: Wijma Delivery Expectancy/Experience Questionnaire.

## Data Availability

The raw data supporting the conclusions of this article will be made available by the authors on request.

## Supplementary Materials

The following supporting information can be downloaded at https://www.mdpi.com/article/10.3390/healthcare13172180/s1: Table S1: PRISMA checklist; Table S2: Search strategies and search terms used in each database.
